# Landlords and Slums

**Published:** 1919-05-31

**Authors:** 


### THE HOUSING BILL.

### II. Landlords and Slums.

The next important section which comes up for consideration is Section 8. This provides, "Where land included in any scheme made under Part I. or Part II. of the principal Act (other than land included in such a scheme only for the purpose of making the scheme efficient and not on account of the sanitary condition of the premises thereon . . .) is acquired compulsorily, the compensation to be paid for the land including any buildings thereon shall be the value . . . of the land as a site cleared of buildings. Provided that in so far as the scheme requires, provision shall be made for the re-housing of persons of the working classes on the site when cleared, the compensation shall be the value . . . of the land as a site for houses for the working classes."

Now in this section there are two subject-matters—namely, compensation for land with insanitary buildings on it, and compensation for land cleared of both insanitary property on it, and of any houses which were included not because they were insanitary, but because they were necessary for the efficient working of the scheme. To understand the effect of this section a short explanation of the position under Parts I. and II. of the principal Act is necessary. When a representation of an insanitary area is made, especially of a large area under Part I. such as the Tabard Street area in Southwark, it is always found that interspersed among the slums and insanitary houses are a certain number of comparatively good houses or groups of houses which cannot be declared as unhealthy, but which nevertheless are so placed that they must be demolished if the space is to be properly opened up and reconstructed. Sometimes there may be a group of quite new houses or a public house or similar building which has been well preserved, while those near by have been allowed to fall into decay, and it is customary to mark these blue on the ord nance map, while the insanitary houses and areas are marked red. Compensation to be paid on those marked blue is ten per cent. more than on those marked red.

The value of the land and houses in the principal Act is the market value at the time of valuation, and of course, it is to the interest of the owners of the land and houses to tender evidence at the inquiry which enhances the value as much as possible. It is true there are numerous provisos to prevent an artificial inflation of the value, but like many safeguards they must be classified among the "paper ones." Any one who has followed an inquiry such as took place over Tabard Street must have been struck not only with the great cost of the insanitary property, but with the strenuous efforts on the part of owners to have their property transferred from red to blue. On the whole, however, what was perhaps more often surprising still was that there was so little opposition to the condemnation of many of the houses. The only conclusions one could draw were that the owners thought they did very well out of the insanitary houses and decided to leave well alone. Broadly speaking, the effect of the provisions in the Act of 1890 was to put a premium on insanitary property and encourage slum landlords to deliberately buy houses if they thought there was any chance of their being condemned under the Housing Acts. If this section is adopted it is likely to put an end to much of the trafficking in slum property, since the value of insanitary houses is completely discounted, and the value of the land is subject to an upper limit if it is going to be used for re-housing. On the other hand, it may make medical officers more chary in condemning areas of houses unless they are bad beyond any dispute, because if the houses be condemned it practically means their confiscation, and owners of such property will, therefore, fight to the bitter end to prove that they are not insanitary or, failing this, that their condition and that of the area can be remedied by less drastic measures than confiscation and total reconstruction. They will go to much trouble and expense to obtain the necessary defence, and it will, therefore, be necessary for the Medical Officer of Health to arm himself with ample corroborative evidence.

There is no doubt, however, that the clauses under question are a step in the right direction. Any enactments which depreciate the value of slum property, and prevent speculation in it, and eventually do away with the slum landlord, are going a long way towards housing and social reform.

The remaining section which confers important additional powers is Section II. This extends the powers of Part III. of the principal Act and enables the local authority to acquire any houses or other buildings on the land intended as a site for working-class houses, and to acquire any estate or interest in any houses which might be made suitable for the working classes. The local authority can improve these houses so as to render them fit for habitation. This is a drastic section and will have the effect of making landlords and owners of property put it and keep it in repair if they do not want it to pass into the hands of the local authority.

Those sections to which special reference has not been made contain various clauses designed to promote the building of dwelling-houses for the working classes by encouragement of public utility societies by making loans and grants, subscribing for shares and loan capital of the society, guaranteeing interest, etc. Other sections deal with relaxation of the present building by-laws under certain circumstances. Penalties are also enacted for letting houses on which closing orders are in force, a very necessary power as any one knows who has experienced the anxiety of many owners to extract the last penny from their slum property.

